# Metabolic Changes in Summer Active and Anuric Hibernating Free-Ranging Brown Bears (*Ursus arctos*)

**DOI:** 10.1371/journal.pone.0072934

**Published:** 2013-09-09

**Authors:** Peter Stenvinkel, Ole Fröbert, Björn Anderstam, Fredrik Palm, Monica Eriksson, Ann-Christin Bragfors-Helin, Abdul Rashid Qureshi, Tobias Larsson, Andrea Friebe, Andreas Zedrosser, Johan Josefsson, My Svensson, Berolla Sahdo, Lise Bankir, Richard J. Johnson

**Affiliations:** 1 Division of Renal Medicine, Department of Clinical Intervention and Technology, Karolinska Institutet, Stockholm, Sweden; 2 Department of Cardiology, University Hospital of Örebro, Örebro, Sweden; 3 Department of Medical & Health Sciences, Experimental Renal Medicine, Linköping University, Linköping, Sweden; 4 Zoologisches Institut, Goethe-Universität, Frankfurt am Main, Germany; 5 Faculty of Arts & Sciences, Department of Environmental & Health Studies, Telemark University College, Porsgrunn, Norway; 6 Institute for Wildlife Biology & Game Management, University for Natural Research & Life Sciences, Vienna, Austria; 7 Department of Nephrology, Aarhus University Hospital, Aarhus, Denmark; 8 Department of Clinical Medicine, School of Health and Medical Sciences, Örebro University, Örebro, Sweden; 9 INSERM Unit 872-E2, Centre de Recherche des Cordeliers, Paris, France; 10 Division of Renal Diseases and Hypertension, University of Colorado Denver, Denver, Colorado, United States of America; University of Sao Paulo Medical School, Brazil

## Abstract

The brown bear (*Ursus arctos*) hibernates for 5 to 6 months each winter and during this time ingests no food or water and remains anuric and inactive. Despite these extreme conditions, bears do not develop azotemia and preserve their muscle and bone strength. To date most renal studies have been limited to small numbers of bears, often in captive environments. Sixteen free-ranging bears were darted and had blood drawn both during hibernation in winter and summer. Samples were collected for measurement of creatinine and urea, markers of inflammation, the calcium-phosphate axis, and nutritional parameters including amino acids. In winter the bear serum creatinine increased 2.5 fold despite a 2-fold decrease in urea, indicating a remarkable ability to recycle urea nitrogen during hibernation. During hibernation serum calcium remained constant despite a decrease in serum phosphate and a rise in FGF23 levels. Despite prolonged inactivity and reduced renal function, inflammation does not ensue and bears seem to have enhanced antioxidant defense mechanisms during hibernation. Nutrition parameters showed high fat stores, preserved amino acids and mild hyperglycemia during hibernation. While total, essential, non-essential and branched chain amino acids concentrations do not change during hibernation anorexia, changes in individual amino acids ornithine, citrulline and arginine indicate an active, although reduced urea cycle and nitrogen recycling to proteins. Serum uric acid and serum fructose levels were elevated in summer and changes between seasons were positively correlated. Further studies to understand how bears can prevent the development of uremia despite minimal renal function during hibernation could provide new therapeutic avenues for the treatment of human kidney disease.

## Introduction

Advanced chronic kidney disease (CKD) is characterized by muscle wasting, cardiovascular disease (CVD), osteoporosis, inflammation and oxidative stress; factors that often occur in combination [1] and herald a poor prognosis [2]. New treatment strategies are urgently needed to decrease the unacceptable high mortality rate in this underserved patient group, but to date most attempts have been disappointing [3].

Animals in the wild live day-to-day as a consequence of evolutionary adaptations that aid their survival [4], including under stressed or extreme conditions. By studying how animals survive extreme conditions, one may identify novel mechanisms that can protect them (i.e. the science of biomimicry). In this context, hibernating free-ranging bears (*Ursidae*) are of special interest to the nephrologist as these amazing creatures adapt up to 6 months of inactivity with anuria and no food or water intake at near normal (30–35°C) body temperatures (in contrast to other hibernating animals) without developing azotemia, muscle wasting or osteoporosis [5]. Their ability to prevent azotemia is a special and (to the best of our knowledge) unparalleled feature of hibernating bears. Humans would not survive even short periods of inactivity and anuria without lethal metabolic complications, such as renal failure or extensive muscle and bone loss [5].

Recently, we have had the opportunity to study free-ranging brown bears (*Ursus arctos*) living in central Sweden, in which we were able to obtain blood samples from the same individuals during their active period in summer and hibernation in winter. We evaluated a number of parameters, including renal function, the calcium-phosphate FGF23 axis, uric acid and fructose levels, and the level and pattern of amino acids in the blood. We present several novel findings in this paper.

## Bears and Methods

### Ethics

No specific permissions were required for research on the locations described in this article as all activities were carried out according to the Swedish Right of Public Access law. The field studies did not involve endangered or protected species. All animal handling and sampling was carried out under approval of the Swedish Ethical Committee on animal research (C212/9) and was in compliance with Swedish laws and regulations. The appropriate authority and ethical committee was “Djuretiska nämnden, Uppsala, Sweden”.

### Bears and Collections of Samples

**Figure 1:**
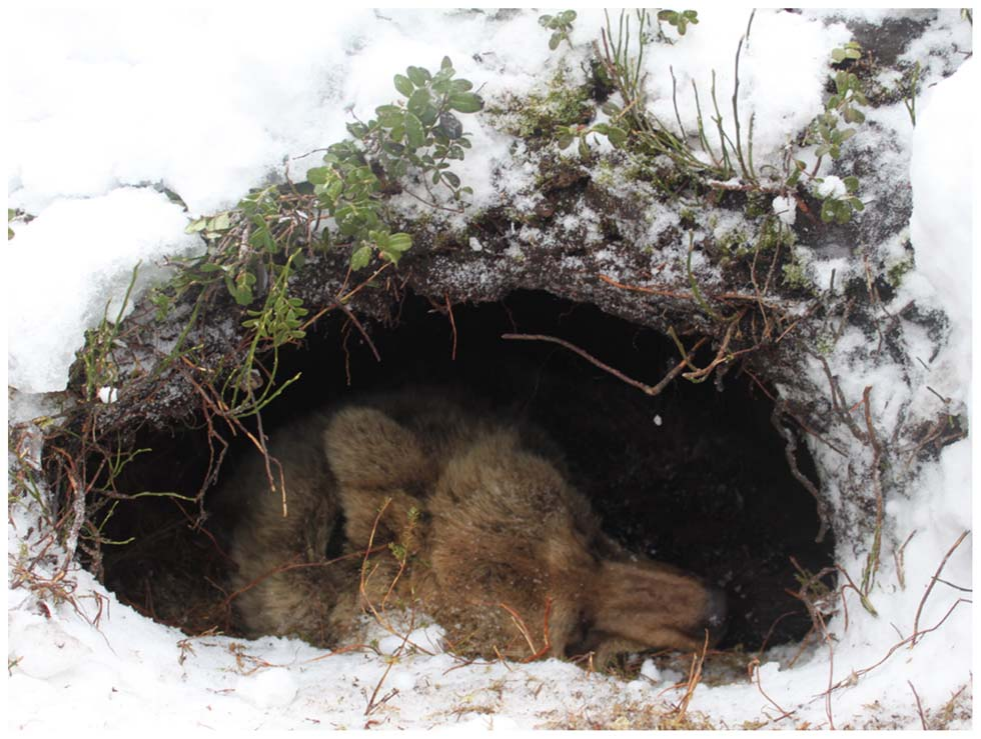
Captured brown bear in its den. Photo: Andrea Friebe, the Scandinavian Brown Bear Research Project.

**Figure 2:**
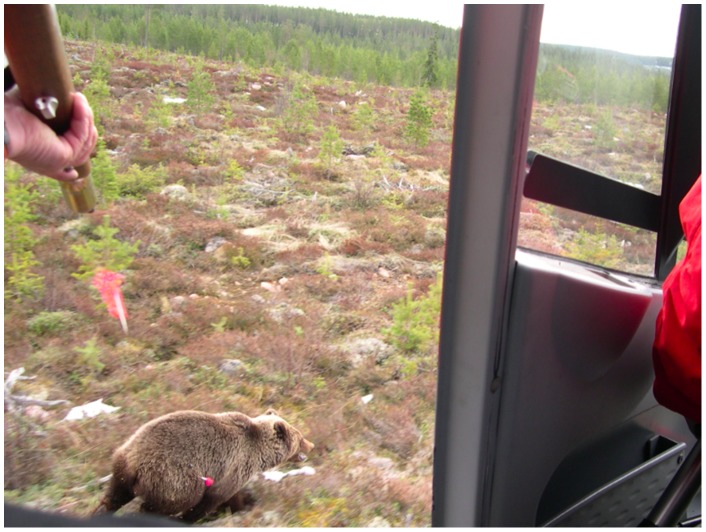
Aerial darting from helicopter of a free-ranging female brown bear during the active summer period. Photo: Andreas Zedrosser, the Scandinavian Brown Bear Research Project.

Samples of blood were taken from 16 free-ranging sub-adult 2- to 3-yr-old Eurasian brown bears [*Ursus arctos*, 11 females and 5 males equipped with a Global Positioning System (GPS) collar with a median body weight of 53 kg (range 22–77 kg)] in Dalarna and Gävleborgs Counties, Sweden, 2010–2012. Each bear was captured during hibernation (February or March) (**Fig. 1**) and during the active period in June of the same year [6]. Bears were immobilized by darting with an anesthetic (see below) in the den and again by darting from a helicopter during June (**Fig. 2**). Bears were weighed on a stretcher suspended beneath a spring scale. In winter the anesthetic consisted of a mixture of tiletamine-zolazepam (1.1 mg/kg), medetomidine (0.03 mg/kg) and ketamine (1.3 mg/kg), and in summer, in a mixture of tiletamine-zolazepam (4.7 mg/kg) and medetomidine (0.09 mg/kg) [6]. In the field, blood samples were taken from the jugular vein within 20 min from darting and collected in tubes containing the additives EDTA, Lithium heparin or clot activator, respectively (Vacuette®; Greiner Bio-one, Frickenhausen, Germany). Approximately 1 hour after sampling blood was centrifuged at 2000xg for 10 min for collection of plasma or serum and immediately frozen on dry ice until storage at −70°C.

### Laboratory Measurements

The following analytes were measured by spectrophotometer with routine methods on a Konelab 20XT centrifugation analyzer (Thermo Fisher Scientific, Vantaa, Finland); albumin with the bromcresol green (BCG) method; glucose using the glucose oxidase/peroxidase Trinder reaction; creatinine with the Jaffe method; cholesterol and triglycerides in coupled enzyme reactions forming quinonimine, respectively; total protein, calcium and phosphorus forming colored complexes with cupric ions, Arsenazo III and ammonium molobdate, respectively; urea using an urease/glutamate dehydrogenase method converting NADH to NAD; and uric acid with a fully automated Trinder (AOX) method using uricase/peroxidase.

Fructose was measured enzymatically with a recently modified sensitive inulin assay [7]. The fructose in the sample is converted to sorbitol using sorbitol dehydrogenase (SDH) and NADH, which is read at 340 nm on the Konelab 20XT pending an incubation for 20 min. The thiol assay measured free sulphydryls in the plasma in a reaction using Ellman's reagent (5,5′-dithio-bis(2-nitrobenzoic acid)) (DTNB). The TNB^2−^ formed was quantified spectrometrically at 412 nm on the Konelab 20XT [8]. Plasma C-reactive protein (CRP) levels were measured by the Immulite Automatic Immunoassay Analyzer (Siemens Medical Solutions Diagnostics, Los Angeles, CA, USA) with an assay manufactured for this analyzer. The assays are not validated for bear plasma. Measurement of pentraxin-3 (PTX3) was accomplished with ELISA's from R&D Systems Inc. (Abingdon, UK). Free plasma amino acids were determined in both human and bear samples by high-performance liquid chromatography with fluorometric detection [9]. Aspartate, cysteine and proline are not possible to analyze for technical reasons. Serum FGF23 was measured using an intact mouse/human FGF23 assay (Kainos, Japan) that only detects the intact, biologically active, protein by using two monoclonal antibodies for capture and detection, respectively. Although this assay has not been developed and/or validated for brown bears, the results yielded physiologically plausible concentrations comparable with those in humans and demonstrated good linearity in diluted samples. In contrast, a C-terminal FGF23 assay and intact FGF23 assay (Immutopics, CA, USA) did not produce detectable FGF23 levels in the current study.

### Statistics

All values are expressed as median (range). Differences between winter and summer samples were analyzed with paired Wilcoxon signed rank test. Comparative statistical between healthy humans and summer bears were analyzed by non-parametric Wilcoxon signed rank test. Changes in the level of some variables between the hibernation and active periods were calculated and shown as “Δ”. Spearman's rank correlation was used to determine correlations between two variables. A p-value <0.05 was considered to be statistically significant. The statistical analysis was performed using statistical software SAS version 9.3 (SAS Campus Drive, Cary, NC, USA).

## Results

The overall sample size was 16 bears in winter and summer, unless stated otherwise. The median body weight in both the active state and during hibernation was 53 kg.

**Figure 3:**
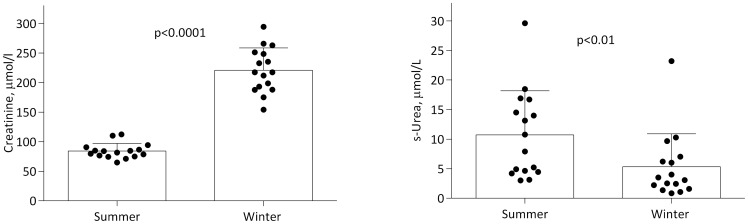
Box plots showing mean and SD as well as individual serum creatinine and urea levels in summer and winter from 16 free-ranging brown bears. The urea/creatinine ratio was about 8 times higher during the active summer period.

Table 1 shows plasma concentrations of different metabolites during summer and winter. The median S-creatinine was 2.5-fold higher during denning than during the active period (p<0.001; Fig. 1), and the median urea concentration was 2-fold lower (p = 0.008; **Fig.** **3**). Thus, the urea/creatinine ratio was markedly higher (p<0.001) during the active state. Whereas no difference in median calcium level was observed between hibernation and the active period (p>0.999), the median serum phosphorous level was significantly lower (p = 0.001) and the median FGF23 significantly higher (p = 0.036) during hibernation than the active period.

**Table 1:** Differences in biochemical and renal parameters between winter and summer samples in 16 free-ranging bears.

	Summer (S)	Winter (W)	W/S ratio	Significance
Weight (kg)	53 (22–77)	53 (21–66)	0.95 (0.79–1.25)	NS
S-creatinine (µmol/L)	83 (65–112)	217 (154–294)	2.64 (1.85–3.53)	p<0.001
Urea (mmol/L)	9.3 (3.0–29.6)	3.3 (0.8–23.2)	0.46 (0.12–1.68)	p<0.01
Urea/creatinine ratio	118 (36–352)	14 (4–124)	0.15 (0.04–0.69)	p<0.001
Calcium (mmol/L)^a^	2.45 (2.27–2.56)	2.44 (2.18–2.55)	1.01 (0.85–1.11)	NS
Phosphate (mmol/L)	1.93 (1.24–2.64)	1.23 (0.53–1.59)	0.59 (0.31–1.26)	p<0.001
FGF23 (pg/ml)^b^	105 (62–532)	203 (164–237)	2.13 (0.39–3.56)	p<0.01
Glucose (mmol/L)	5.6 (2.5–10.8)	7.5 (5.4–12.5)	1.14 (0.72–4.52)	p<0.05
Fructose (µmol/L)	138 (89–287)	99 (39–383)	0.69 (0.25–1.61)	p<0.05
Uric acid (µmol/L)	96 (29–299)	48 (33–120)	0.58 (0.16–4.22)	p<0.05
Cholesterol (mmol/L)	6.4 (3.7–8.8)	10.4 (8.0–18.9)	1.56 (1.21–2.59)	p<0.001
Triglycerides (mmol/L)	2.3 (0.9–3.3)	4.8 (2.0–7.0)	2.19 (1.08–3.59)	p<0.001
Total protein (g/L)	57.5 (49.6–68.3)	72.5 (47.8–80.9)	1.25 (0.96–1.47)	p<0.001
Albumin (g/L)	28.3 (22.5–31.8)	36.2 (31.3–43.8)	1.24 (1.13–1.69)	p<0.001
Thiols (µmol/ L)^c^	267 (239–284)	471 (372–472)	1.67 (1.44–1.97)	p<0.05
CRP (µg/L)^d^	4.5 (0.0–11.2)	6.0 (1.3–13.2)	1.03 (0.42–7.43)	NS
PTX3 (ng/ml)	0.07 (0.01–0.38)	0.08 (0.05–0.13)	1.08 (0.31–10.0)	NS

Median and range.

a n = 10, ^b^n = 13, ^c^n = 5, ^d^ normal value for humans <2 mg/L.

**Figure 4:**
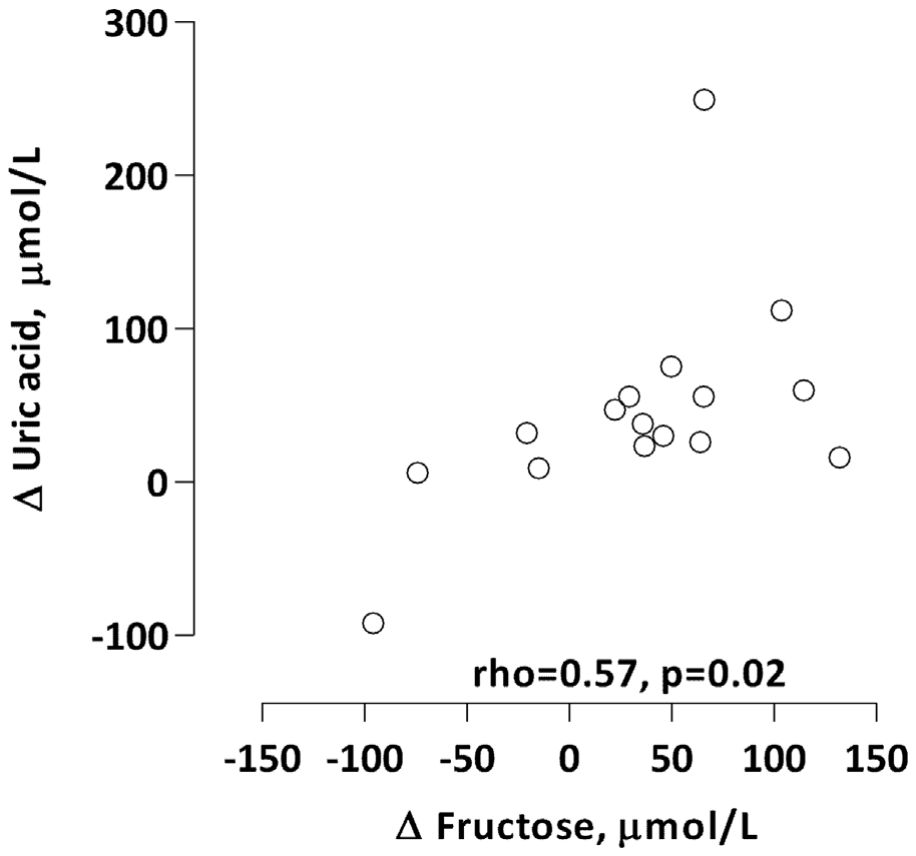
A positive correlation was observed between changes (Δ) in fructose and uric acid from winter to summer. This suggest that higher uric acid levels observed during the active summer period is in part dependent on increased fructose intake via fruits and berries.

The glucose level was significantly higher during hibernation than the active period (p = 0.039). Both, the median fructose (p = 0.041) and the uric acid (p = 0.03) levels were significantly lower during hibernation than the active period. We found a significant positive correlation between the levels of fructose and uric acid during hibernation (ρ = 0.66, p = 0.007) but not during the active period (ρ = −0.18, p = 0.498). However, the changes in the levels of fructose and uric acid between hibernation and active period (Δfructose and Δuric acid) correlated significantly and positively (ρ = 0.57, p = 0.021; **Fig. 4**). Both the median cholesterol (p<0.001) and triglyceride levels (p<0.001) were significantly higher during hibernation than the active period (**Table 1**). Also, the total protein (p<0.001) and S-albumin (p<0.001) levels were significantly higher during hibernation. A significant positive correlation was observed between Δalbumin and Δcholesterol (ρ = 0.63, p = 0.009). The correlation between Δtotal protein and Δalbumin was significant and positive (ρ = 0.83, p<0.001). Whereas no significant differences in the levels of either CRP (p = 0.231) or PTX3 (p = 0.307) were observed between the hibernation and active period, higher levels of free thiols was observed during hibernation (p = 0.043). Due to the low number of bears (n = 5) this p-value should be interpreted with caution.

Median levels of amino acids in the active summer and hibernating winter periods are given in Table 2 and were compared to those of healthy humans. The median concentration of total amino acids was significantly higher in bears during summer than in humans. This is mostly due to a 37% higher concentration of essential amino acids (EAA) and a modest (although significant) 3% higher concentration of non-essential amino acids (NEAA). Note that EAA and NEAA are listed in Table 2 according to the amino acid requirements known for humans. No differences in the median level of total, EAA, NEAA or branched chain amino acid (BCAA) concentrations in bears were observed between hibernation winter and the active summer period (**Table 2**). However, this apparent stability is the consequence of very diverse pattern of changes in the concentration of individual amino acids (**Table 2**). Of note, several amino acids did not change significantly between hibernation and active summer period. This is the case for all three BCAAs (leucine, isoleucine and valine). Tryptophane, phenylalanine, glycine, serine and alanine also showed no change between the two periods. In contrast, some amino acids were significantly higher (lysine, histidine, 3-methylhistidine, glutamine and glutamic acid) while others were significantly lower (threonine, methionine, asparagine, tyrosine and taurine) during hibernation than the active summer period (**Table 2**). Arginine and its precursor citrulline exhibited opposite changes. Amino acids that are involved in nitrogen excretion, i.e., glutamine (for the synthesis of ammonia) and ornithine (for the synthesis of urea), exhibited coordinated increases.

**Table 2:** Amino acid levels during summer and winter in 15 sub-adult free-ranging bears and 39 healthy subjects (28 males) with age 68 years (range 38–80 years).

		Healthy Subjects	Bears: Summer	Bears: Winter	Significance^c^
**Total amino acids (µmol/L)**		2587 (1504–3852)	2887 (2068–3454)^b^	3041 (2606–3913)	NS
**Total EAA (µmol/L)**		699 (434–957)	959 (555–1145)^b^	999 (816–1309)	NS
**Total NEAA (µmol/L)**		1886 (1069–2894)	1945 (1447–2675)^b^	2055 (1775–2604)	NS
**Total BCAA (µmol/L)**		379 (246–531)	445 (178–576)	430 (309–561)	NS
Leucine (µmol/L)	EAA	109 (64–155)	146 (60–212)^b^	135 (95–189)	NS
Isoleucine (µmol/L)	EAA	53 (26–75)	69 (30–103)^b^	74 (58–107)	NS
Valine (µmol/L)	EAA	216 (151–307)	225 (89–281)	210 (155–268)	NS
Tryptophan (µmol/L)	EAA	44 (28–66)	42 (23–61)	39 (24–55)	NS
Phenylalanine (µmol/L)	EAA	52 (38–67)	58 (37–79)^b^	65 (40–88)	NS
Glycine (µmol/L)	NEAA	215 (125–391)	285 (167–591)^b^	302 (195–362)	NS
Alanine (µmol/L)	NEAA	313 (126–609)	599 (276–735)^b^	440 (324–699)	NS
Serine (µmol/L)	NEAA	88 (60–148)	98 (66–147)	88 (59–127)	NS
Lysine (µmol/L)	EAA	148 (89–219)	191 (61–3219)^a^	345 (254–424)	p<0.001
Histidine (µmol/L)	EAA	79 (48–120)	88 (31–137)	106 (68–149)	p<0.05
3-metylhistidine (µmol/L)	NEAA	missing	11 (4–29)	37 (23–54)	p<0.001
Glutamine (µmol/L)	NEAA	633 (364–1012)	502 (317–743)^b^	733 (570–917)	p<0.001
Glutamic acid (µmol/L)	NEAA	33 (9–89)	57 (41–70)^b^	66 (43–115)	p<0.05
Threonine (µmol/L)	EAA	129 (49–224)	158 (73–192)^a^	117 (80–149)	p<0.01
Methionine (µmol/L)	EAA	22 (12–43)	46 (28–77)^b^	25 (817–34)	p<0.001
Aspargine (µmol/L)	NEAA	43 (22–60)	37 (21–49)^a^	22 (4–34)	p<0.001
Tyrosine (µmol/L)	NEAA	60 (36–108)	54 (835–79)	38 (26–48)	p<0.001
Taurine (µmol/L)	NEAA	40 (22–62)	143 (61–260)^b^	100 (27–118)	p<0.05
Arginine (µmol/L)	NEAA	79 (39–129)	112 (32–230)^a^	80 (54–100)	p<0.05
Ornithine (µmol/L)	NEAA	38 (5–93)	33 (12–54)	71 (41–120)	p<0.001
Citrulline (µmol/L)	NEAA	40 (25–75)	40 (20–145)	59 (48–81)	p<0.05

Median and range, NS; not significant, ^c^ winter vs summer.

BCAA; branched chain amino acids; EAA; essential amino acids; NEAA; non-essential amino acids.

Differences between healthy subjects versus summer levels of 15 sub-adult bears are denoted with ^a^p<0.05, ^b^p<0.001.

## Discussion

In this paper we present a series of metabolic measurements on free-ranging brown bears during hibernation in comparison to the active period. To our knowledge, this study represents the largest series to date that investigates nitrogen metabolism, the calcium-phosphate axis, and measurements of nutrition indices (lipids, glucose, amino acids and uric acid) in free-ranging bears in two metabolically different periods of the year. Several interesting novel findings were observed.

### Nitrogen metabolism

This study confirms the remarkable ability of the brown bear to recycle urea nitrogen during the hibernation period. As mammals cannot hydrolyze urea (because they do not possess the enzyme urease) it has to be excreted by the kidney. A previous study in bears has shown that glomerular filtration rate (GFR) is reduced to about only one fourth of its normal value during hibernation (from 122 to 37 ml/min) [10]. As the bladder becomes leaky so that water, electrolytes and nitrogen wastes are returned to the blood, bears are anuric during the entire hibernation [11]. Despite the decrease in GFR and the recycling of urine, serum urea is lower during hibernation than during the active period due to the unique ability of bears to recycle urea nitrogen back to protein [12]. In contrast to the lower urea concentration, creatinine (another endproduct of protein metabolism) was 2.5-fold higher during hibernation than during summer. Creatinine, the endproduct of creatine, which is the high-energy storage molecule in muscle, is not metabolized and is cleared only by the kidney. Since bladder urine returns to the blood, creatinine cannot be excreted during hibernation and accumulates in the blood. However, this elevation remains relatively modest because creatinine generation is probably reduced in winter due to the lack of muscle activity. In most cases, when kidney function is reduced in humans and other mammals, serum creatinine and urea concentrations usually rise in parallel. In the case of hibernation, we observe a remarkable divergence in serum urea and creatinine (**Fig. 3**) that is explained by the possibility to re-use urea nitrogen, but not creatinine, through symbiotic gut bacteria.

The mechanism(s) responsible for the reduction in urea during hibernation probably involves a combination of factors. First, a reduction in metabolic rate probably leads to lesser urea synthesis in the liver. During winter bears obtains most of their energy from metabolizing stored fat producing only CO_2_ and H_2_O as end products. Second, as the urea that is still produced is recycled back into skeletal muscle and other body proteins [13] it has been speculated that (like in ruminants and some other herbivorous mammals) urea is hydrolyzed by urease-expressing gut bacteria into ammonia and CO_2_. Ammonia is then used by enterocytes to synthetize glutamine which may be incorporated into proteins [14]. In ruminants, a facilitated urea transporter is expressed in the rumen epithelium [15]. A similar urea transporter is also expressed in the colon of other mammals [16] including in humans [17]. Most likely, bears express a similar urea transporter in the colon and/or some subsegment of the intestinal wall. Ahlqvist et****al [18] showed that by serving as a carbon source for amino acid formation, glycerol released from fats might help prevent azotemia during winter denning. The observation by Nelson et****al [19] that urea levels decrease in the autumn, when food is still available, suggests that metabolic changes occur prior to the hibernation state. This occur as a consequence of increased intake of fruits and berries prior to hibernation [20]. As berries provide abundant carbohydrates with little proteins, it reduces the need to synthetize and excretes urea.

### Nutritional Parameters

Bears increase their food intake in the late summer and autumn to increase their fat stores before hibernation, and then will survive hibernation primarily by burning these fat stores [21]. In this study, the hibernating bears showed evidence for enhanced fat stores as noted by the higher levels of serum triglycerides and cholesterol levels (**Table 1**), which confirms findings by Arinell et****al [22]. We observed higher levels of both the total protein and albumin in hibernation samples. This may be due to a modest dehydration during hibernation because bears (although inactive) cannot prevent some water loss through the airways in the expired air. However, this finding also indicates preserved protein stores and confirms Lohuis et****al [23], who showed that protein synthesis and breakdown were in balance during winter anorexia. As bears spare most of their crucial skeletal muscle proteins during hibernation anorexia, it seems conceivable that reserves from other organs may be used as sources for nitrogen during denning. Complicated protein kinetic studies of other organ metabolism, such as liver and kidney, are needed to resolve this issue.

Several mammals, including hibernating marmots (*Marmota*) and ground squirrels (*Sciuridae*) as well as long distance migratory birds, develop insulin resistance in preparation for the period of fasting associated with hibernation or migration [24], [25]. In this study the hibernating bears had slightly higher glucose levels suggesting the presence of insulin resistance. In many hibernating animals, the brain might utilize the release of ketones, such as β-hydroxybutyric acid instead of glucose during hibernation [26]. Whether this is also occurring in bears is not known. In the present study we also noted a two-fold higher uric acid level in the active period in comparison to hibernation. Serum uric acid of hibernating mammals tends to be elevated during the active period and to decrease during torpor [27]. Nelson et****al [12] studied two captive American black bears (*Ursus americanus*) and noted higher urinary uric acid excretion in the autumn before hibernation. Previous studies in hibernating squirrels have shown that there is a rapid drop in both inosine and uric acid in the liver during hibernation [28], [29], consistent with an inhibition of AMP deaminase. We have recently found evidence that AMP deaminase activity is low in the liver of the 13-lined ground squirrel *(Ictidomys tridecemlineatus*) during hibernation (R Johnson, unpublished) and a decrease in AMP deaminase may be important for the activation of AMP kinase and the burning of fat [30]. In addition, higher uric acid levels in summer could represent the generation of uric acid during the metabolism of purines and fruits. Fruits contain fructose, a monosaccharide that generates uric acid during its catabolism [31]. Fructose has been shown to increase fat stores in a variety of animals as well as to induce insulin resistance [32], and the mechanism is likely mediated in part by the effect of uric acid to induce mitochondrial oxidative stress [33]. For bears, fruit intake increases particularly in the autumn where it is a food source used to help fatten the animal. The typical bear diet during late summer and autumn includes enormous amount of berries, such as from the *Vaccinum* family [i.e. bilberries (*V. myrtilus*), huckleberrries (*V. parvifolium*) and lingonberries (*V. vitis-idea*)], which are rich in both fructose [34] and resveratrol [35]. It has been reported that large bears in captivity can eat as much as 260,000 huckleberries/day when fed *ad libitum* [36]. In fact, in a feeding and foraging trial using captive and wild American black bears, maximum intake ranged from 30 g/min for berries to amazing >200 g/min for fruits [20]. It should also be taken into account that during the ripening process of berries during the summer and autumn their content of fructose, flavonol, abscisic acid (a plant hormone) and anthocyanins increases [37] whereas vitamin C content decreases [38]. Considering the enormous amount of berries consumed by bears in the summer and autumn, their effects on metabolism and renal function need further studies.

### Amino acids

We demonstrated higher total amino acid, EAA and NEAA levels in summer active bears compared to healthy humans. Interestingly, more than 3-fold higher taurine levels were observed in bears during their active period. Since taurine is key for the conjugation of the unique bear bile ursodeoxycholic acid it has been speculated that taurine deficiency in captive bears causes metabolic bone disease due to decreased absorption of vitamin D [39]. Previous studies on bears have shown discrepant results regarding changes in plasma amino acid levels; with constant [12], elevated [40] or decreased [41] levels reported during winter anorexia. Our amino acid analyses demonstrated no seasonal change in either total amino acids, EAA, NEAA or BCAA levels. This contrasts with the dramatic changes in amino acid levels that occur during prolonged starvation in humans [42], deep hibernators, such as hedgehogs (*Erinaceinae* spp) [43], and other mammals undergoing prolonged fasting, such as elephant seals (*Mirounga* spp*)* [44]. This suggests that bears maintain a remarkably stable whole amino acid balance despite several months of anorexia and inactivity. As inflammation contributes to low amino acid levels in CKD patients [45] the absence of inflammation during hibernation may contribute to maintained amino acid levels in bears.

Several specific changes in individual amino acid concentrations were observed during hibernation (**Table 2**). For the following discussion we assume that EAAs are identical in bears and humans. Although this has not been evaluated to our knowledge, a recent study suggests that the essential amino acids are the same in humans, rats, dogs and a few other species, with differences only in quantitative requirements among species [46]. In the present study, five of the EAAs did not change significantly between summer and winter. Since there is no food intake and since these AAs are assumed not to be synthetized in the body, it means that their balance is kept constant, and thus, that they are not at all degraded (since they could not be re-synthetized, being “essential”). BCAA (leucine, isoleucine and valine) usually serve as metabolic fuel in muscle and kidney. During hibernation, the metabolism is markedly reduced and the level of these amino acids is, thus, unchanged. It is notable that methionine, a sulfur amino acid, declined to almost half of its summer value. The increased concentration of lysine and histidine may result from some protein breakdown. The possibility that amino acids synthetized by the gut microflora might be used by the bears require further studies.

A larger fraction of the NEAAs showed seasonal changes since only three out of 12 did not change significantly. The three NEAA that exhibit a similar concentration during hibernation as during the active period are the three smallest amino acids (glycine, alanine and serine). Interestingly, the amino acids that are involved in nitrogen excretion in the form of urea and ammonia exhibit coordinated changes in winter. We observed about 50% higher levels of glutamine during hibernation compared to the active summer state. As glutamine is formed from glutamate and ammonium (NH_4_^+^) by glutamine synthase, increased glutamine formation may provide a mechanistic explanation to counter unwanted NH_4_^+^ buildup during conditions of reduced renal clearing capacity. Glutamine is the amino acid that serves to transport nitrogen between organs. Notably, glutamine is used to transfer nitrogen from the digestive tract to the kidney where NH_4_ is produced and excreted [47]. Furthermore, glutamine exerts translational control of mitochondrial uncoupling protein (UCP)-2 by binding to an open reading frame [48] and UCP-2 mediates mitochondrial uncoupling by facilitating proton translocation across the mitochondrial inner membrane without the production of ATP [49]. Interestingly, increased UCP-2 activity results in defective glucose-induced insulin release from pancreatic beta-cells [50], which could explain the apparent hyperglycemia during hibernation. It is tempting to propose that the increased glutamine concentration during hibernation is a mechanism to signal increased mitochondrial uncoupling, and thus increased heat production, from peripheral tissue other than the UCP-1 containing brown adipose tissue. The normally devastating UCP-2-mediated increased kidney oxygen usage, resulting in intrarenal hypoxia and kidney disease [51], is likely prevented by the low GFR, and thus low oxygen demand, during hibernation. The observation that both glutamine and ornithine are significantly increased in winter also suggests a lower metabolic rate of these nitrogen excretion-related pathways. During normal feeding, dietary arginine is almost entirely degraded in the liver. The body's supply in arginine originates from the synthesis of arginine in the kidney, using citrulline that is released by the liver in the blood stream [52]. The opposite differences observed during hibernation vs. summer in citrulline (increase) and arginine (decline) is consistent with a reduced metabolic activity of the kidney.

### Calcium-Phosphate Axis

In humans, long-term inactivity, as well as a reduction in kidney function, are associated with substantial changes in parameters of bone and mineral metabolism [5]. In contrast, a striking feature in bears is that calcium levels (as observed in the present study) and bone mineral density are unaltered during hibernation, indicating the presence of bone-preserving mechanisms [53], [54]. In this study, we found that serum inorganic phosphorous level was significantly lower during hibernation. This may reflect a state of starvation and reduced caloric intake with redistribution from extracellular to intracellular space when cellular phosphate demand is insufficient. This has previously been demonstrated in healthy humans and in CKD patients with protein energy wasting and/or reduced protein intake [55]. Low winter phosphate levels may also reflect bone-preserving mechanisms; i.e. no excess phosphate is released from bone. As renal and intestinal phosphate transport is modulated by amino acids and glucose it is possible that also other metabolic changes will off-set downstream alterations in phosphate metabolism. Although numerous studies in man and rodents support that FGF23 is stimulated by dietary phosphate intake and hyperphosphatemia [56], [57], [58], we observed higher levels of circulating FGF23 during hibernation, despite the presence of hypophosphatemia. Similarly, there is a gradual increase in FGF23 that parallels the decline in kidney function in patients with CKD [56], [59]. Thus, these data are consistent with the hypothesis that GFR *per se* is a strong regulator of FGF23, independent of serum inorganic phosphorous level or dietary phosphate intake.

### Inflammation

In humans reduced renal function is commonly associated with low-grade persistent inflammation [60]. However, in the present study circulating levels of the inflammatory biomarkers CRP and PTX3 were markedly low both during hibernation and the active period (**Table 1**), which indicate that despite reduced renal function and prolonged inactivity bears do not develop systemic inflammation during hibernation. This may be due to a documented increase in antioxidant levels during hibernation, especially increases in vitamin C levels [61]. The high levels of vitamin C may act as a mitochondrial antioxidant and counter the effects of uric acid, thereby aiding the oxidation of fat [27], [33]. It could also be speculated that high intake of resveratrol via *Vaccinum* berries may contribute to the antioxidative milieu in bears [62]. We observed markedly higher levels of taurine of bears, which also may contribute to their overall anti-oxidant defense [63]. Notably, taurine depletion is a common feature of the pro-oxidant human uremic milieu [9]. Other possibilities include the effect of low body temperature in blocking the immune system [64], changes in plasma bile acid composition, such as ursodeoxycholic acid [65] known to have anti-inflammatory effects [66], or changes in mitochondrial function and the regulation of apoptosis [67], [68].

### Strengths and limitations

The present study is, to our knowledge, the largest study to date on free-ranging bears. Sixteen bears were each studied twice while living in their normal environment and fed *ad libitum* their natural diet. Several coordinated metabolic pathways and markers were studied simultaneously (carbohydrate, lipid and nitrogen metabolism, calcium-phosphate balance and inflammation). The small number of sampled bears limited most previous studies and the majority was conducted under laboratory or enclosure conditions. Such studies may not mimic the natural circannual fattening cycle and eating habits as well as the natural winter hibernation profiles. However, some limitations remain. First, the analytic methods were developed for human assays and the accuracy for bear samples could not be unequivocally determined. Secondly, due to limited amount of serum/plasma, some analyses could not be performed in all bears. Three proteinogenic amino acids are missing in our analyses and we did not measure, hematocrit, plasma sodium and osmolality (which could have given information about the hydration status of the animals). It should also be acknowledged that as sampling was performed in February (or March) and June, respectively, our data are not representative of late hibernation or late summer-autumn conditions. Although body weight did not differ between hibernation and the active summer period significant differences in body composition would probably be observed if bears were instead captured at the end of the hibernation period (i.e. leanest) and late autumn (i.e. fattest). As the bear diet changes in the late summer when berries are abundant and start to ripe, it is possible that dietary changes may lead to a different metabolic milieu. Indeed, a previous study indicated that urea levels decline by about 50% between summer and autumn samples [19], which indicates that changes in the bear diet may reduce the intensity of nitrogen catabolism even before the fasting period.

## Conclusions

In conclusion, the present study of 16 free-ranging brown bears confirms that despite anuria and 2.5-fold increase in creatinine levels, azotemia does not develop. While total EAA, NEAA and BCAA concentrations do not change during hibernation anorexia, changes in individual amino acids ornithine, citrulline and arginine indicate an active, although reduced urea cycle and nitrogen recycling to proteins. Changes in glutamine and citrulline are consistent with a reduced metabolism in the kidney, in agreement with the reduced GFR. The reasons and implications for the 2–3 fold higher levels of taurine and alanine in bears needs further studies. We also find elevated FGF23 but lower phosphorous levels during hibernation. Despite prolonged inactivity and reduced renal function, inflammation does not ensue and bears seem to have enhanced antioxidant defense mechanisms during hibernation. The significant decrease in uric acid levels despite reduced renal functions indicates a reduced nucleic acid catabolism during hibernation. Further studies of these metabolic magicians may lead to novel interventions for both prevention and treatment of uremic complications.
